# Effects of fermented soybean meal supplementation on the growth performance and apparent total tract digestibility by modulating the gut microbiome of weaned piglets

**DOI:** 10.1038/s41598-023-30698-6

**Published:** 2023-03-06

**Authors:** Madesh Muniyappan, Sureshkumar Shanmugam, Jae Hong Park, Kyudong Han, In Ho Kim

**Affiliations:** 1 Department of Animal Resource and Science, Dankook University, Cheonan-si, Chungnam 31116 South Korea; 2 Department of Microbiology, College of Science and Technology, Dankook University, Cheonan, 31116 South Korea; 3 Center for Bio Medical Engineering Core Facility, Dankook University, Cheonan, 31116 South Korea

**Keywords:** Microbiology, Gastroenterology

## Abstract

This study investigates the effects of soybean meal fermented by *Enterococcus faecium* as a replacement for soybean meal on growth performance, apparent total tract digestibility, blood profile and gut microbiota of weaned pigs. Eighty piglets (weaned at 21 days) [(Landrace × Yorkshire) × Duroc] with average body weight of 6.52 ± 0.59 kg) were selected and assigned to 4 treatments/4 replicate pens (3 barrows and 2 gilts). The four diets (SBM, 3, 6 and 9% FSBM) were formulated using fermented soybean meal to replace 0, 3, 6 and 9% of soybean meal, respectively. The trial lasted for 42 days phase 1, 2 and 3. Result showed that supplemental FSBM increased (*P* < 0.05) the body weight gain (BWG) of piglets at day 7, 21 and 42 and ADG at days 1–7, 8–21, 22–42 and 1–42, and ADFI at days 8–21, 22–42 and 1–42 and G: F at days 1–7, 8–21 and 1–42, and crude protein, dry matter, and gross energy digestibility at day 42, and lowered (*P* < 0.05) diarrhea at days 1–21 and 22–42. The concentration of glucose levels, WBC, RBC, and lymphocytes were increased while, concentration of BUN level in the serum was lowered in the FSBM treatment compared to the SBM group (*P* < 0.05). Microbiota sequencing found that FSBM supplementation increased the microbial Shannon, Simpsons and Chao indexs, (*P* < 0.05) and the abundances of the phylum *Firmicutes,* and genera *prevotella, Lactobacillus*, *Lachnospiraceae* and *Lachnoclostridium* (*P* < 0.05), lower in the abundances of the phylum *bacteroidetes*, *Proteobacteria*, genera *Escherichia-Shigella*, *Clostridium sensu stricto1*, *Bacteroides* and *Parabacteroides* (*P* < 0.05). Overall, FSBM replacing SBM improved the growth performance, apparent total tract digestibility, and blood profiles; perhaps via altering the faecal microbiota and its metabolites in weaned pigs. The present study provides theoretical support for applying FSBM at 6–9% to promote immune characteristics and regulate intestinal health in weaning piglets.

## Introduction

In the swine husbandry, weaning produce gut system dysfunctions and results from inconstant impairment of the gut barrier function, oxidative stress and absorption as a result of poor growth, diarrhoea and other diseases^1,2^. Weaning pigs are immediately required to undergo a change from high digestible sow’s milk to solid diets as well as complex protein^3,4^. Therefore, it is important to explore potential protein sources that should be added to diets to alleviate the weaning stress of piglets. The high cost, finite supply, and unstable variation of sources animal protein had become major reasons for limiting its supplement in diets on weaning pigs. Soybean meal (SBM) is an important protein feed ingredient in livestock diet, but variety of antinutritional factors (ANF) as well as β-conglycinin, glycinin and trypsin inhibitor, which would lead to immune responses, digestive disorders and negative effects on animal health^3,5^.

Probiotics are alternatives to in-feed antibiotics because they are successful in improving livestock production, efficiency and welfare^6^. Enterococcus faecium is a gram-positive gamma-hemolytic or non-hemolytic bacterium in the genus Enterococcus is a kind of facultative anaerobic lactobacillus that can colonize the gastrointestinal tract of humans and animals^7^. Enterococcus faecium bacterium feeds have shown positive effects in piglets intestinal microbiota, increasing immunoreactivity while reducing diarrhea^8^. The administration of living microbial preparations is one part of probiotic treatments. While, the another effective pathway is microbial fermentation of feed. Cheng et al.^9^ and Li et al.^10^ reported that bacterial fermentation could reduce content by ANFs and improving on nutritional quality and nutrient bioavailability. Fermented soybean meal (FSBM), a manufactured product mixed with solid SBM, liquid phases and the vaccinating the mixture with *E. faecium*^11^ could improve protein quality and reduce on ANFs levels of SBM with solid state fermentation^12^. It has been reported that FSBM has partial or total replacement of SBM to improve the growth performance and apparent tract total digestibility (ATTD) of crude protein and gross energy immune and antioxidant capacity in weaning pigs^2^. Jeong et al.^13^ found that compared with SBM, FSBM had improve the growth performance and the apparent ileal digestibility of crude protein, dry matter, and gross energy and amino acids in weaning piglets. Moreover, compared with SBM, FSBM showed greater concentration of crude protein and amino acid and reduce trypsin inhibitor and ANFs^14^.

The ideal situation would be to select feed probiotics that would improve soybean meal quality and enhance resistance to weaning stress by boosting the gut microbiota. Using *Enterococcus faecium (E. faecium)* toproduce a fermented soybean meal (FSBM) in weaning pigs has not been stated earlier and a probiotic-fermented SBM might show some different effects on piglets. The objective of the current study was to compare the effects of dietary SBM and FSBM on growth performance, ATTD, blood profile and gut microbiota of weaning pigs.

## Materials and methods

### Animal care

The animal care and experimental procedures described in this experiment were conducted according to the Animal Welfare Committee guidelines and had the approval of the Ethics committee of Animal Resource and Science College of Dankook University (DK-2-1936, Cheonan, South Korea). And the experiments were performed in accordance with the Animal Research: Reporting of In Vivo Experiments (ARRIVE) guidelines (https://arriveguidelines.org).

### Preparation of fermented soybean meal (FSBM)

The Enterococcus faecium SLB130 were grown in de Man, Rogosa and Sharpe (MRS) medium at 37 °C. Fermentation was initiated by soaking SBM in distilled water to a achieve 30% moisture content. Water-soaked SBM and inoculated with a 10% of Enterococcus faecium SLB130 to achieve 10^6^ cfu/g in SBM. SBM mixtures were anaerobically solid-state fermented at 37 °C for 48 h using a previously published protocol^15^. Finally, the fermented SBM was dried at 50–60 °C to a moisture concentration of 10% and then ground in a hammer mill. Final fermented SBM consisted of 5.8 × 10^7^/g. Crude protein, KOH protein solubility of FSBM was determined by official methods of analysis^16^. Glycinin, β-conglycinin and trypsin inhibitor in FSBM were tested using a commercial kit (Feed Up Co., Ltd., Republic of Korea) (Table 1).

**Table 1:** Compositions of soybean meal (SBM) and fermented soybean meal (FSBM).

Item	SBM	FSBM
Crude protein (%)*	33.13 ± 0.43^b^	39.78 ± 0.26^a^
Metabolizable energy**, kcal/kg	3732	3720
Crude fiber, %	4.00 ± 96	4.08 ± 56
Crude Ash, %	5.56 ± 0.29	5.84 ± 0.084
KOH protein solubility (%)	86.69 ± 0.51^a^	75.45 ± 1.83^b^
TCA soluble protein (%)	2.21 ± 0.06^b^	11.06 ± 0.08^a^
Glycinin (mg/g)*	140.22 ± 0.08^a^	28.88 ± 1.33^b^
β-conglycinin (mg/g)*	113.42 ± 1.49^a^	36.13 ± 0.29^b^
Trypsin inhibitor (mg/g)*	11.16 ± 0.4^a^	0.33 ± 0.02^b^
Stachyose (%)	4.57 ± 0.057^a^	0.18 ± 0.03^b^
Raffinose (%)	2.81 ± 0.16^a^	0.54 ± 0.03^b^

*On a dry matter basis; ^a,b^, Means within rows with different letters differed significantly (P < 0.05). The comparison was conducted in a horizontal manner.

**Metabolizable energy of SBM, FSBM is measured.

### Experimental design, animals and feeding method

Eighty [(Landrace × Yorkshire) × Duroc] crossed weaned piglets (21 of age; 6.52 ± 0.59 kg) were randomly selected and allocated into 4 diets with 4 pens replicates according to the average initial body weight and sex, and each pen has 5 piglets (3 barrows and 2 gilts). Four diets were formulated using FSBM to replace 0, 3, 6 and 9% of SBM, respectively. The trial lasted for 42 days (phase 1 (days 1–7), phase 2 (8–21) and phase 3 (days 22–42). Basal diets were formulated to meet the NRC (2012) requirements (Tables 2, 3 and 4). Throughout the trial, all pigs had free access to feed and water, and the room temperature was maintained at 24–26 °C with 60–70% humidity, respectively.

**Table 2:** Composition of weaning pig diets (as fed-basis).

Item	Dietary treatments^a^ Phase 1 (1–7)			
	FSBM0	FSBM3	FSBM6	FSBM9
Ingredients (%)				
Corn	39.29	39.89	39.67	39.45
Soybean meal	22.18	20.06	17.64	15.22
FSBM	–	3.00	6.00	9.00
Spray-dried plasma protein	6.00	6.00	6.00	6.00
Tallow	0.78	1.36	1.02	0.69
Coconut powder (60% fat)	2	–	–	–
Lactose	13.46	13.46	13.46	13.46
Sugar	2.00	2.00	2.00	2.00
Whey protein	11.00	11.00	11.00	11.00
Monodicalcium phosphate	1.40	1.38	1.38	1.38
Limestone	0.94	0.94	0.94	0.94
Salt	0.10	0.10	0.10	0.10
Methionine (99%)	0.19	0.18	0.18	0.17
Lysine	0.23	0.20	0.18	0.16
Mineral mixture^b^	0.20	0.20	0.20	0.20
Vitamin mixture^c^	0.20	0.20	0.20	0.20
Choline (25%)	0.03	0.03	0.03	0.03
Total	100.00	100.00	100.00	100.00
Calculated chemical composition				
Crude protein, %	20.00	20.00	20.00	20.00
Metabolizable energy, kcal/kg	3400	3400	3400	3400
Calcium, %	0.80	0.80	0.80	0.80
Available phosphorus, %	0.70	0.70	0.70	0.70
Lysine, %	1.40	1.40	1.40	1.40
Methionine, %	0.45	0.45	0.45	0.45
FAT, %	3.82	3.72	3.95	4.2
Lactose, %	20.00	20.00	20.00	20.00

^a^FSBM0, FSBM3, FSBM6 and FSBM9 diets were made by substituting fermented soybean meal for 0, 3, 6 and 9% soybean meal.

^b^Provided per kg diet: Fe, 100 mg as ferrous sulfate; Cu, 17 mg as copper sulfate; Mn, 17 mg as manganese oxide; I, 0.5 mg as potassium iodide; and Se, 0.3 mg as sodium selenite.

^c^Provided per kilograms of diet: vitamin A, 10,800 IU; vitamin D3, 4000 IU; vitamin E, 40 IU; vitamin K3, 4 mg; vitamin B1, 6 mg; vitamin B2, 12 mg; vitamin B6, 6 mg; vitamin B12, 0.05 mg; biotin, 0.2 mg; folic acid, 2 mg; niacin, 50 mg; D-calcium pantothenate, 25 mg.

**Table 3:** Composition of weaning pig diets (as fed-basis).

Item	Dietary treatments^a^ Phase 2 (8–21)			
	FSBM0	FSBM3	FSBM6	FSBM9
Ingredients (%)				
Corn	54.75	55.34	55.1	54.91
Soybean meal	27.42	25.30	22.88	20.42
FSBM	–	3.00	6.00	9.00
Spray-dried plasma protein	1.50	1.50	1.50	1.50
Tallow	2.73	3.31	2.99	2.65
Coconut powder (60% fat)	2.00	–	–	–
Lactose	3.89	3.89	3.89	3.89
Sugar	2.00	2.00	2.00	2.00
Whey protein	2.00	2.00	2.00	2.00
Monodicalcium phosphate	1.58	1.56	1.56	1.56
Limestone	0.98	0.99	0.99	0.99
Salt	0.10	0.10	0.10	0.10
Methionine (99%)	0.17	0.16	0.16	0.16
Lysine	0.45	0.42	0.4	0.39
Mineral mixture^b^	0.20	0.20	0.20	0.20
Vitamin mixture^c^	0.20	0.20	0.20	0.20
Choline (25%)	0.03	0.03	0.03	0.03
Total	100.00	100.00	100.00	100.00
Calculated chemical composition				
Crude protein, %	19.50	19.50	19.50	19.50
Metabolizable energy, kcal/kg	3400	3400	3400	3400
Calcium, %	0.80	0.80	0.80	0.80
Available phosphorus, %	0.70	0.70	0.70	0.70
Lysine, %	1.40	1.40	1.40	1.40
Methionine, %	0.45	0.45	0.45	0.45
FAT, %	6.28	6.19	6.44	6.67
Lactose, %	4.98	4.98	4.98	4.98

^a^FSBM0, FSBM3, FSBM6 and FSBM9 diets were made by substituting fermented soybean meal for 0, 3, 6 and 9% soybean meal.

^b^Provided per kg diet: Fe, 100 mg as ferrous sulfate; Cu, 17 mg as copper sulfate; Mn, 17 mg as manganese oxide; I, 0.5 mg as potassium iodide; and Se, 0.3 mg as sodium selenite.

^c^Provided per kilograms of diet: vitamin A, 10,800 IU; vitamin D3, 4000 IU; vitamin E, 40 IU; vitamin K3, 4 mg; vitamin B1, 6 mg; vitamin B2, 12 mg; vitamin B6, 6 mg; vitamin B12, 0.05 mg; biotin, 0.2 mg; folic acid, 2 mg; niacin, 50 mg; D-calcium pantothenate, 25 mg.

**Table 4:** Composition of weaning pig diets (as fed-basis).

Item	Dietary treatments^a^ Phase 3 (22–42)			
	FSBM0	FSBM3	FSBM6	FSBM9
Ingredients (%)				
Corn	59.12	59.7	59.47	59.24
Soybean meal	28.32	26.18	23.76	21.34
FSBM	–	3.00	6.00	9.00
Tallow	2.00	2.60	2.27	1.94
Coconut powder (60% fat)	2.00	–	–	–
Lactose	2.69	2.69	2.69	2.69
Sugar	2.00	2.00	2.00	2.00
Monodicalcium phosphate	1.64	1.64	1.64	1.64
Limestone	0.98	0.98	0.98	0.98
Salt	0.10	0.10	0.10	0.10
Methionine (99%)	0.17	0.16	0.16	0.16
Lysine	0.55	0.52	0.50	0.48
Mineral mixture^b^	0.20	0.20	0.20	0.20
Vitamin mixture^c^	0.20	0.20	0.20	0.20
Choline (25%)	0.03	0.03	0.03	0.03
Total	100.00	100.00	100.00	100.00
Calculated chemical composition				
Crude protein, %	19.00	19.00	19.00	19.00
Metabolizable energy, kcal/kg	3350	3350	3350	3350
Calcium, %	0.80	0.80	0.80	0.80
Available phosphorus, %	0.70	0.70	0.70	0.70
Lysine, %	1.40	1.40	1.40	1.40
Methionine, %	0.45	0.45	0.45	0.45
FAT, %	5.72	5.64	5.88	6.12
Lactose, %	2.50	2.50	2.50	2.50

^a^FSBM0, FSBM3, FSBM6 and FSBM9 diets were made by substituting fermented soybean meal for 0, 3, 6 and 9% soybean meal.

^b^Provided per kg diet: Fe, 100 mg as ferrous sulfate; Cu, 17 mg as copper sulfate; Mn, 17 mg as manganese oxide; I, 0.5 mg as potassium iodide; and Se, 0.3 mg as sodium selenite.

^c^Provided per kilograms of diet: vitamin A, 10,800 IU; vitamin D3, 4000 IU; vitamin E, 40 IU; vitamin K3, 4 mg; vitamin B1, 6 mg; vitamin B2, 12 mg; vitamin B6, 6 mg; vitamin B12, 0.05 mg; biotin, 0.2 mg; folic acid, 2 mg; niacin, 50 mg; D-calcium pantothenate, 25 mg.

### Growth performance

Each piglet was weighed on days 0, 7, 21 and 42 and feed consumption was also recorded on a pen basis to determine average daily gain (ADG), average daily feed intake (ADFI) and gain to feed ratio (G:F = ADG/ADFI).

### Diarrhea score

The piglets’ anuses were checked one by one at 09:00 and 17:00 daily during the experiment to observe and recorded any fecal contamination and redness method by Ma et al.^17^. The number of piglets with diarrhea per treatment was counted at the days 1–21 and 22–42 and the diarrhea rate was calculated with the following formulation:

$$ {\text{Diarrhea}}\;{\text{rate }}\left( \% \right) = {1}00\% \times {\text{total}}\;{\text{number}}\;{\text{of}}\;{\text{piglets}}\;{\text{with}}\;{\text{diarrhea}}/({\text{total}}\;{\text{number}}\;{\text{of}}\;{\text{piglets}} \times {\text{number}}\;{\text{of}}\;{\text{days}}). $$

### Apparent total tract digestibility

To determine dry matter, crude protein, and gross energy digestibility, chromium oxide was added to the diet as an indigestible marker at 2 g/kg of the diet for7 day prior to fecal collection. Fecal samples were collected from 8 pigs randomly selected per treatment via rectal massage, and the sample was stored in a freezer at − 20 °C and was dried in a 65 °C for 72 h and the feed and fecal samples were grounded to passed through 1-mm sieve for the measurement of dry matter, crude protein, and gross energy of FSBM, SBM, diets and feces samples were determined following to the Association of Official Analytical Chemists^18^ procedures.

### Blood profile

At 06:00 on days 21 and 42, two pigs for each replicate was randomly selected to collect blood from the jugular vein and subsequently centrifuged at 3,000 × g for 15 min at 4 °C to obtain the serum sample and was kept at − 80 °C until analysis by Muniyappan et al.^19^ The concentrations of lymphocyte counts, Red blood cells (RBC), White blood cell (WBC), Blood urea nitrogen (BUN), and glucose in serum were measured with an automatic biochemical analyzer (Model 7020, Hitachi, Tokyo, Japan). The serum creatinine concentration was determined using anAstra-8 analyzer (Beckman Instruments, Inc., Brea, CA, US).

### Characteristics of microbial population in feces

On day 42, fresh fecal samples were collected from six pigs for microbiota analysis. Genomic DNA of fecal samples was extracted by using a DNA Kit (Omega Bio-tek, Norcross, GA, USA), according to manufacturer’s instructions. The quantity and quality of extracted genomic DNA were checked using a UV spectrophotometer (Mecasys, Daejeon, Korea). Amplification and sequencing of the V5–V6 hypervariable region of the 16S rRNA gene was performed using an Illumina MiSeq platform (Illumina, San Diego, CA, USA). We performed alpha-diversity and taxonomic analyses of the raw paired-end sequences using EZBioCloud pipeline^20^. Then the samples were grouped into microbiome taxonomic profile sets for further analyses. Relative abundance cut-offs at the phylum and genus levels were set to 0.1%. Charts depicting the results from the alpha-diversity and taxonomic analyses were generated using GraphPad Prism software version 9.0 (GraphPad, San Diego, California, USA).

### Statistical analysis

All results are presented as mean ± standard deviation (SD). GraphPad 9.0 was used for figures. Statistical analyses were performed using IBM SPSS 20.0 (SPSS Inc., Chicago, IL, USA) and the differences between treatments were compared with one-way ANOVA followed by Dunnett’s multiple comparison procedure. For all tests, *P* < 0.05 was considered as significant.

## Results

### Growth performance

**Figure 1:**
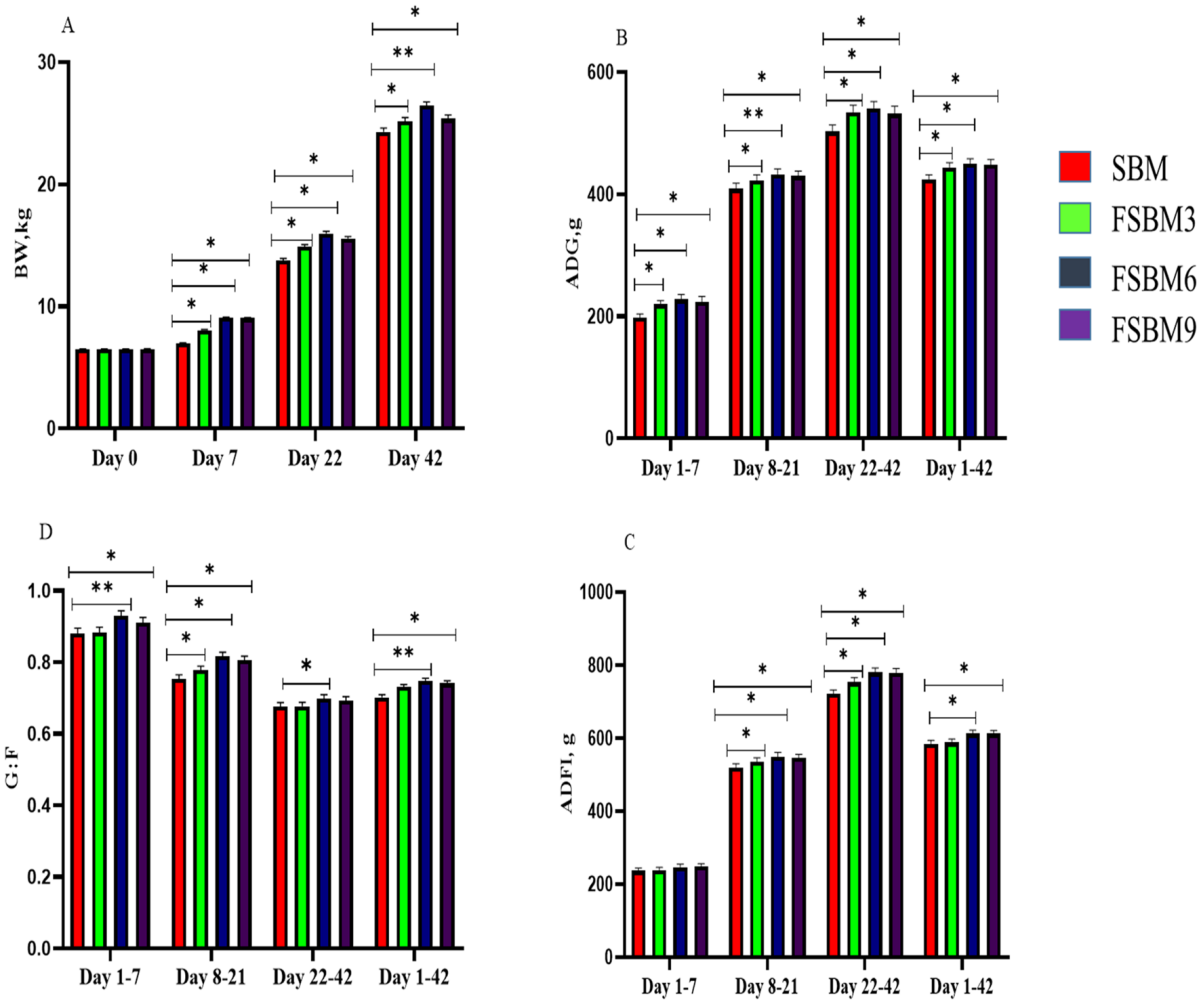
Effect of fermented soybean meal (FSBM) on growth performance in piglets. (**A**) Body weight (BW). (**B**) Average daily gain (ADG). (**C**) Average daily feed intake (ADFI). (**D**) Gain-to-feed ratio (G:F). Data are presented as means ± SD. *P < 0.05, **P < 0.01. SBM, soybean meal; FSBM, fermented soybean meal.

As shown the Fig. 1, the BW of piglets fed dietary FSBM was higher than SBM. The 3% and 6% FSBM (*P* < 0.05) during days 7, 21 and 42, 9% FSBM (*P* < 0.05) during days 7 and 22, (*P* < 0.01) during day 42 all showed significant increases. Compared with SBM treatments, piglets fed 3, 6 and 9% FSBM (*P* < 0.05) on the higher ADG, ADFI and G:F during days 1–7, 8–21, 22–42 and 1–42. Importantly, the diarrhea rate lower in FSBM treatments (1–21, 22–42). (*P* < 0.05).

### Diarrhea score

**Figure 2:**
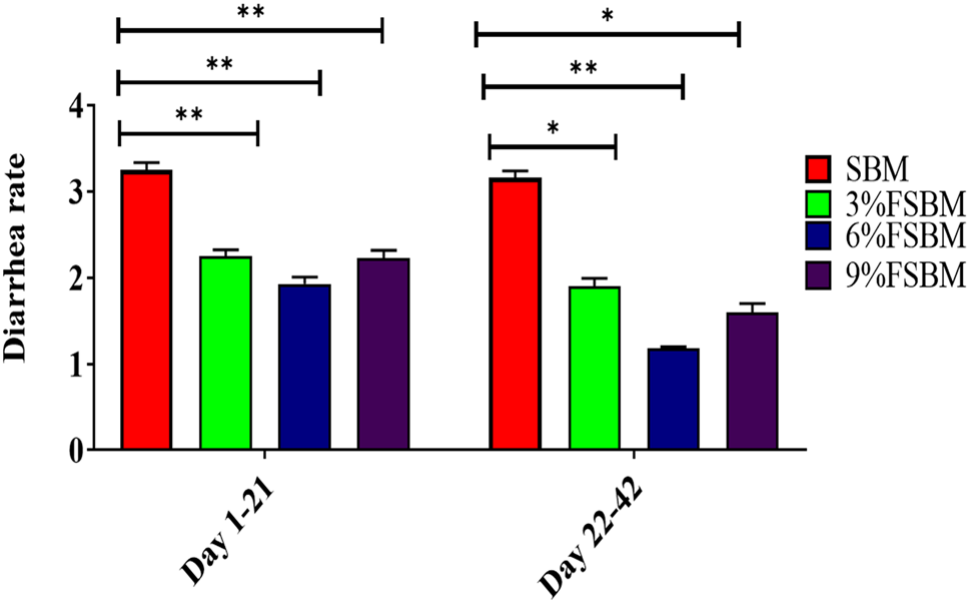
Effect of fermented soybean meal (FSBM) on diarrhea rate in piglets. Data are presented as means ± SD. *P < 0.05, **P < 0.01. SBM, soybean meal; FSBM, fermented soybean meal.

Piglets fed FSBM diets had significantly lower (*P* < 0.05) the diarrhea score on days 1–21 and 22–42 (Fig. 2).

### Apparent total tract digestibility

**Figure 3:**
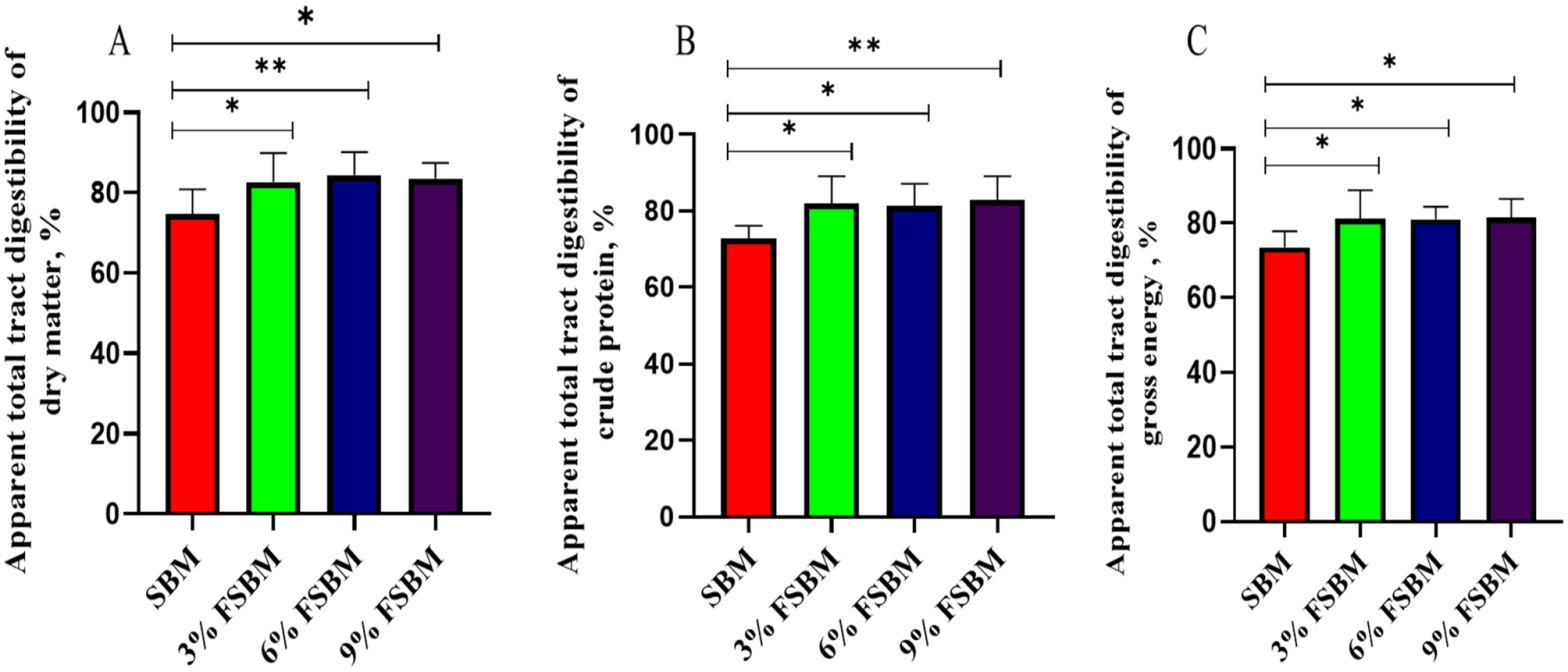
Effect of fermented soybean meal (FSBM) on apparent total tract digestibility in piglets. (**A**) Dry matter. (**B**) Crude protein. (**C**) Gross energy. *P < 0.05, **P < 0.01. SBM, soybean meal; FSBM, fermented soybean meal.

Dietary supplementation with 3, 6 and 9% FSBM increased (*P* < 0.05) the ATTD of dry matter, crude protein, and gross energy compared to the SBM (Fig. 3). Higher (*P* < 0.05) dry matter was found in the piglets dietary supplemented with 6% FSBM than 3 and 9% FSBM. However, 9% FSBM diet showed elevated crude protein levels than piglets fed 3 and 6% FSBM.

### Blood profile

**Figure 4:**
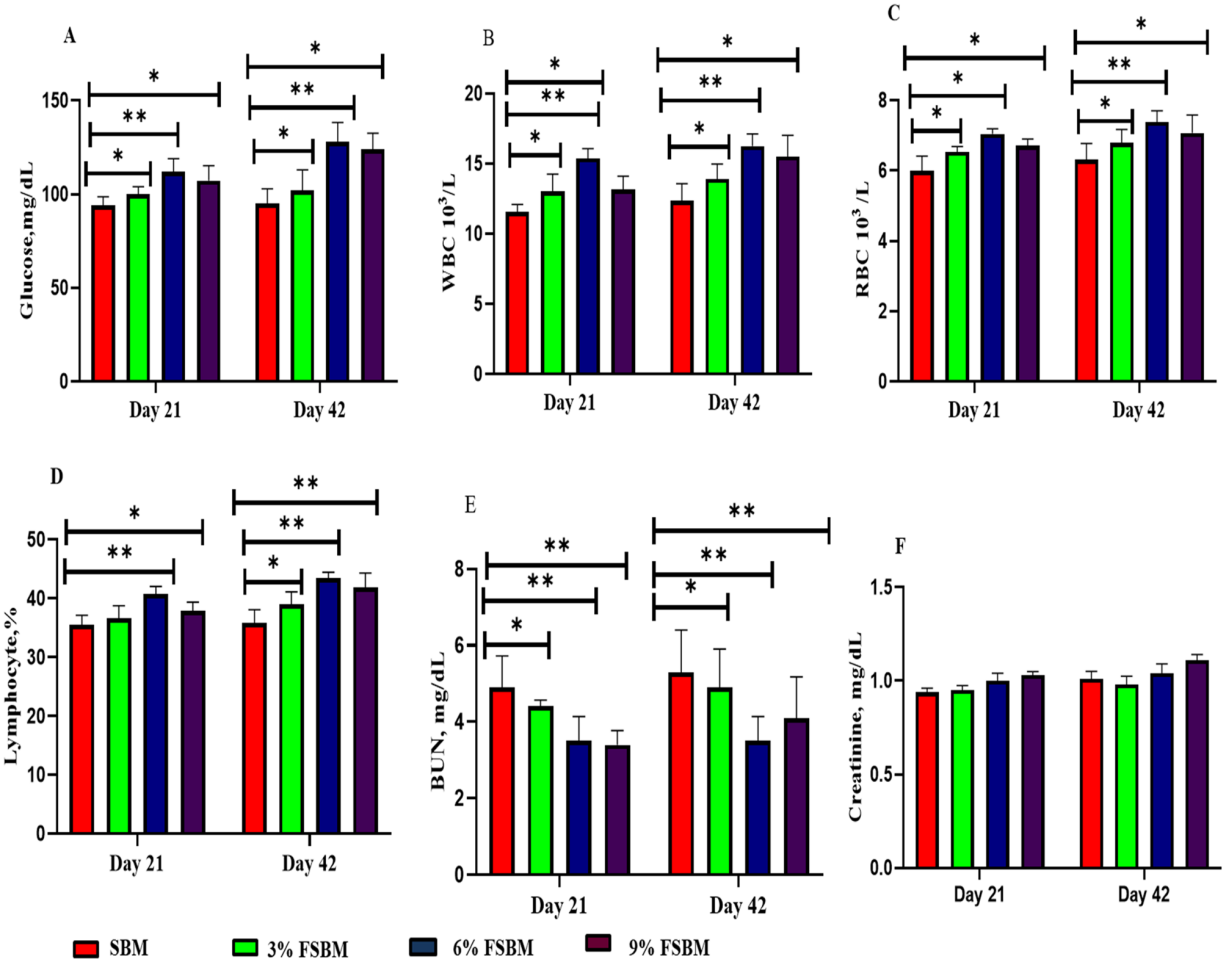
Effect of fermented soybean meal (FSBM) on blood profiles in piglets. (**A**) Glucose levels. (**B**) White blood cell (WBC). (**C**) Red blood cell (RBC). (**D**) Lymphocyte. (**E**) Blood urea nitrogen (BUN). (**F**) Creatinine. *P < 0.05, **P < 0.01. SBM, soybean meal; FSBM, fermented soybean meal.

As illustrated in Fig. 4, piglets diets FSBM diets had increased (*P* < 0.05) the glucose levels, WBC, RBC, and lymphocytes and lowered (*P* < 0.05) the BUN level on days day 21 and 42. High (*P* < 0.05) glucose, RBC, WBC, Lymphocyte, and lower BUN compared with SBM was observed (Fig. 3) in serum gathered from days 21 and 42 piglets supplemented with the 6% FSBM. BUN was lower in the latter two group than in 3 and 6% FSBM. The Creatinine was not influenced in the serum by feeding 3, 6 and 9%FSBM.

### Effects of FSBM on fecal microbial composition

**Figure 5:**
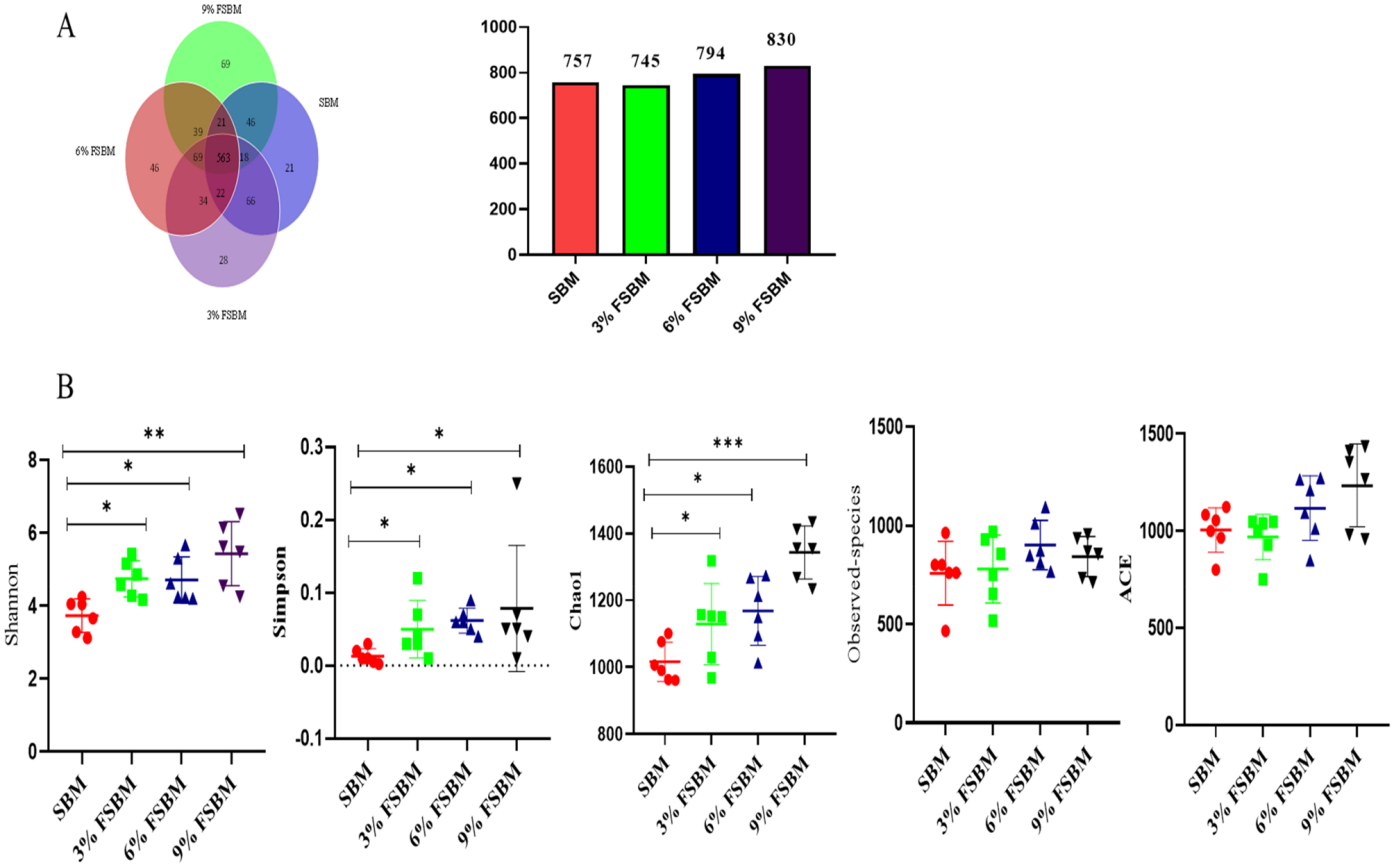
Fecal microbial richness and diversity. (**A**) Venn diagrams of dietary treatments at the OTUs levels. (**B**) Microbial richness estimates (Observed species, Chao, and Ace) and diversity indices (Shannon and Simpson). *P < 0.05, **P < 0.01. SBM, soybean meal; FSBM, fermented soybean meal.

The OTUs Venn analysis identified 21, 28, 46 and 69 unique OTUs among FSBM and SBM treatments, respectively (Fig. 5A). Figure 4B illustrates the results of alpha diversity analysis of which the Shannon, Simpsons and Chao indices were improved (*P* < 0.05) on FSBM compared to the SBM (Fig. 5B).

**Figure 6:**
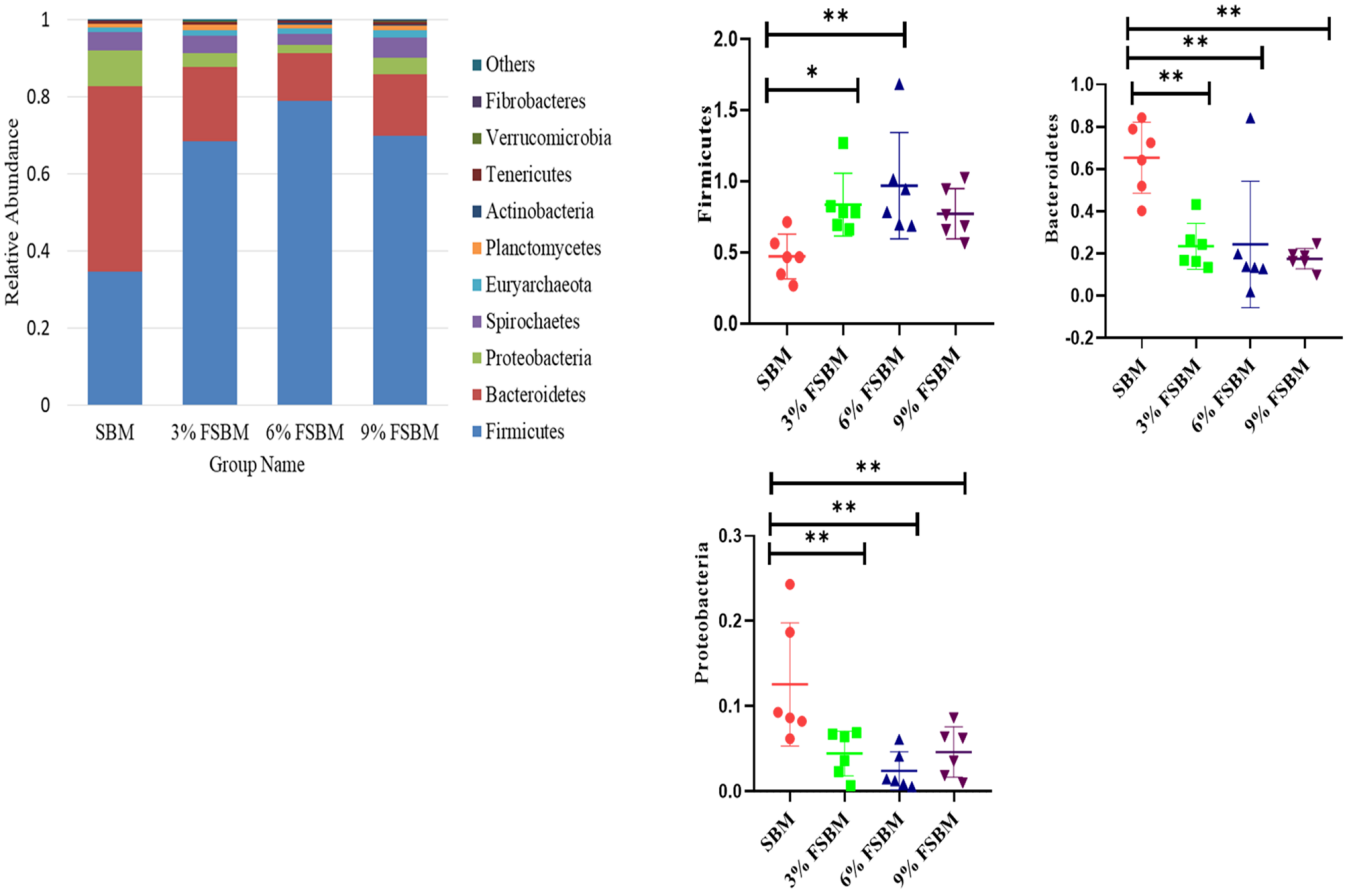
Effects of dietary FSBM on fecal microbial composition at the phylum level. *P < 0.05, **P < 0.01, and ***P < 0.001. SBM, soybean meal; FSBM, fermented soybean meal.

**Figure 7:**
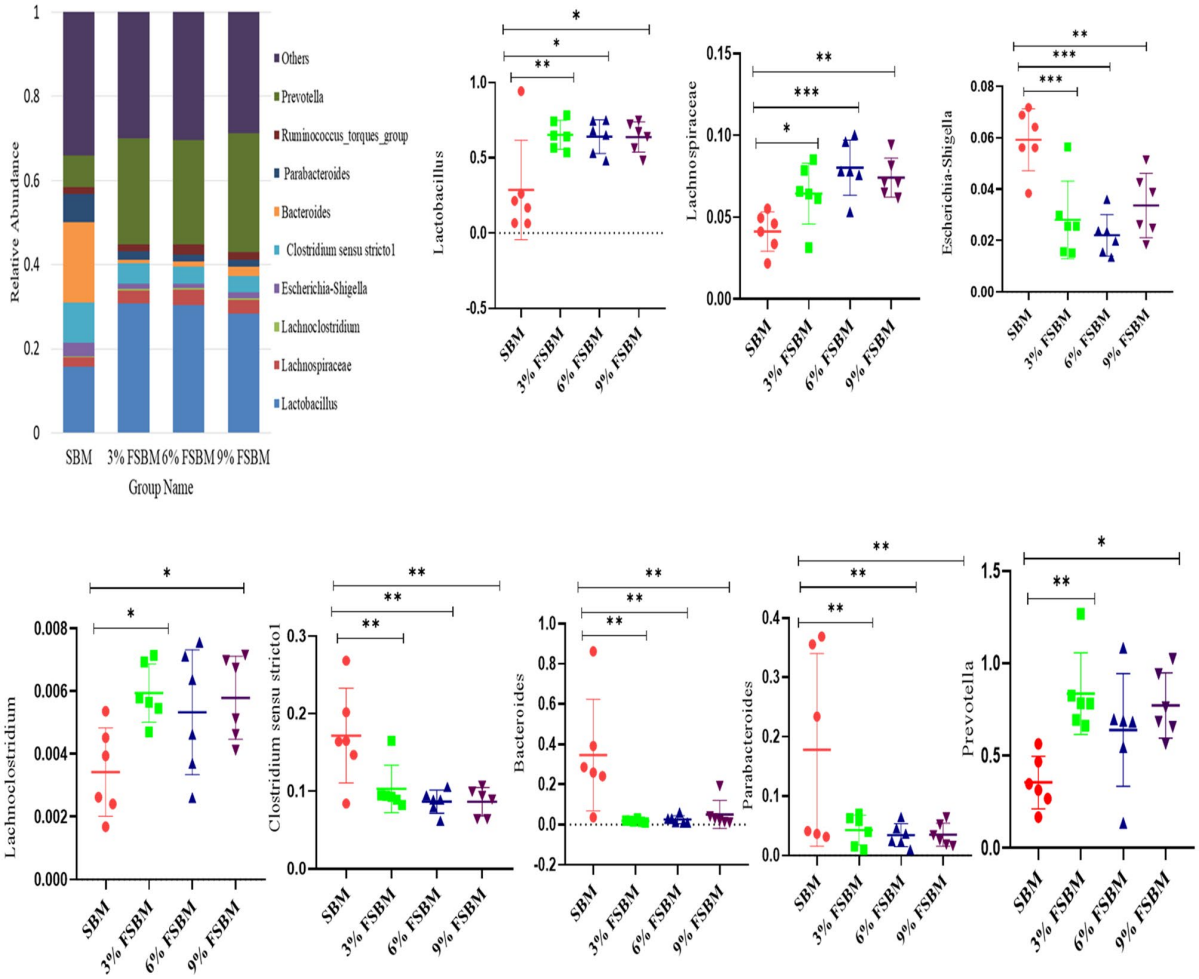
Effects of dietary FSBM on fecal microbial composition at the genus level. *P < 0.05, **P < 0.01, and ***P < 0.001. SBM, soybean meal; FSBM, fermented soybean meal.

As shown in Fig. 6, the phylum levels analysis illustrated that the dietary supplementation of FSBM higher in the abundance of *Firmicutes* (*P* < 0.05) and lower the abundance of Bacteroidetes and *Proteobacteria* (*P* < 0.05). At the genus level, FSBM group increased *Lactobacillus, prevotella, Lachnospiraceae* and *Lachnoclostridium* (*P* < 0.05) in fecal microbiota (*P* < 0.05). Compared with SBM treatment, FSBM treatments lower in the abundance of *Escherichia-Shigella, Clostridium sensu stricto1, Bacteroides and Parabacteroides* (*P* < 0.05) (Fig. 7).

## Discussion

### Growth performance and diarrhea score

Sources of plant protein like FSBM have been extensively used in the diets of weaning pigs to enhance growth and immune status^2,4^. Earlier studies, illustrated that dietary FSBM supplementation increased the growth performance on weaning pigs^21–23^ and broilers^9^. Zhu et al.^24^ reported a significantly in the increased growth performance in weaning piglets due to dietary FSBM supplementation. In current study, the BW, ADG, and ADFI were improved in weaning pigs supplemented with FSBM treatments. Trypsin inhibitor content and antigenic protein content was reduced to 0.33 and 20%, respectively. Stachyose and raffinose were completely absent in FSBM. Pigs fed with FSBM could improve nutrient digestibility that may explain an enhanced ADG in weaning pigs.

### Diarrhea score

The positive effect of SBM substitution by FSBM on diarrhea reduction may be attributed to the degradation of the main antigenic soybean proteins such as α and α′ subunits of β-conglycinin and acidic subunits of glycinin and some antinutritional factors such as trypsin inhibitors^25,26^. These proteins are partially digested by proteases secreted by microorganisms responsible for the fermentation process^27,28^. Similarly, Jeong et al.^11^ showed that degradation of most antigenic proteins (glycinin and β-conglycinin) and protease inhibitors in SBM fermented by *Enterococcus Faecium* improved intestinal morphology and digestive enzyme activities in weaned pigs. Fermentation of soybeans also degrades its proteins and carbohydrates to the extent of low molecular weight and water-soluble compounds that facilitate their digestibility^29^. The combination of a better nutritional status and a reduced immunological challenge when pigs are fed FSBM, or FSB may help to prevent diarrhea after weaning. Previous study, we demonstrated that dietary FSBM inclusion reduce the diarrhea in weaning pigs^24,30^. In the current study, piglets fed FSBM decreased the diarrhea in weaning pigs. In addition, it has been proposed that some microorganisms present in fermented products can inhibit intestinal colonization of pathogens that causes diarrhea in pigs^31^. The reduction of diarrhea is of great importance in pig production as it decreases the predisposition of these animals to *Escherichia coli* infection and improves feed efficiency, especially in weaning pigs^32^.

### Apparent total tract digestibility

In the current study, supplementation of FSBM significantly increased digestibility of crude protein, dry matter, and gross energy which was consistent with Yan and Kim^33^ and Muniyappan et al.^15^ who reported that FSBM inclusion increased apparent total tract of crude protein, dry matter, and gross energy in weaning pigs. The beneficial effects of FSBM on nutrient digestibility may be attributed to the effects of FSBM on gastrointestinal development and the concomitant increase in digestive enzyme secretion^4^. Similarly, Min et al.^34^ found that dietary FSBM did not have significant effect on the digestibility of dry matter, crude protein, and gross energy in weaning pigs. In contrast, the negative effects of crude protein, dry matter, and gross energy on nutrient digestibility were also observed in previous studies^13,35^. Similarly, Hossain et al.^36^ and Lan and Kim^37^ found that dietary inclusion of FSMB did not have a significant effect on the digestibility of crude protein, dry matter, and gross energy in weaning pigs. The inconsistent results may be related to many factors such as FSBM source, additional levels, and composition of the basal diet, with effects being more pronounced when moderate amounts of FSBM are added to SBM basal diets^21,34^.

### Blood profile

The BUN concentration and producing are affected by way of protein catabolism, and its concentrating is negatively associated with digestibility of proteins and amino acids^38^. Creatinine exists a natural waste result arises from the muscles and is eliminated from the body through kidney. Pigs fed FSBM showed decreased diarrhea score and the glucose levels improved which was in accordance with earlier studies^24,39^. Intriguingly, serum blood urea nitrogen reduced in FSBM treatments compared with the control. This shown that the fermenting procedure change nitrogen distribution inside the feed^40^. The levels of white blood cells, lymphocytes, were also increased, in the current studies. Lymphocyte growth exists as a major phase during the immune reaction in an animal and a proliferative reaction is a specific antigen^41^. Gizzarelli et al.^42^ and Wang et al.^43^ reported that weaning pig immunity is lower on β-conglycinin is not adequate deactivated during fermentation. Our results found a higher immune resistance on piglets this directly corresponded with a decrease in glycinin (80.29%) and β-conglycinin (69.43%) on FSBM. Therefore, overall growth performance and blood profiles were in accordance with each other.

### Effects of FSBM on fecal microbial composition

Diets offer available substrates inclusive of protein carbohydrates for the gut microbiota and affect microbial structure and metabolism might encourage the performance and gut health of weaned pigs^2^. Generally, dietary nutrients are absorbed and digested in the foregut, later undigested food residues and endogenous are fermented by gut microbiota in the hindgut^44,45^. Fermentation of carbohydrates is enhanced to colonic cells due to the production of SCFA^2,45^, during fermentation of indigestible protein manufacture potentially metabolite toxicity inclusive of Ammonia, amines, phenols, and indoles that have harmful effects on intestinal health^44^. Alpha diversity can be utilized as an indicator of the functional resilience of ecological diversity of the intestinal microbiota, including species richness indices (Observed species, Chao, and Ace), and species diversity (Shannon and Simpson)^46^. In the current study, dietary inclusion with FSBM significantly increased the Shannon, Simpson, and Chao1 richness, which was in accordance with our previous findings in pigs that dietary inclusion of FSBM significant difference the Shannon, Simpson^47^. *Bacteroidetes, Firmicutes, Actinobacteria, Proteobacteria, and Tenericutes* were the most pre-dominant bacteria phylum in the piglets^48^. In the current study, FSBM group shapes gut microbiota in weaning pigs, including lower in the abundances of the phylum *Bacteroidetes,* and *Proteobacteria* and the genera *Escherichia-Shigella, Clostridium sensu stricto1, Bacteroides and Parabacteroides*, and a higher in the abundances of the phylum *Firmicutes* and the genera *Lactobacillus, prevotella, Lachnospiraceae* and *Lachnoclostridium*. *Bacteroidetes, Proteobacteria and Firmicutes* as three major communities, are essential to growth performance and energy metabolism homeostasis^49,50^. Shin et al.^51^ found a lower in the abundance of the phylum *Proteobacteria* in the gut of healthy humans. Therefore, Litvak et al.^52^ reported that increase in *Proteobacteria* abundance have been associated with in humans with gut inflammation, colorectal cancer, irritable bowel syndrome and metabolic syndrome and could be bacterial signature of gut dysbiosis^51^. The abundance of the phylum *Proteobacteria* contains many possible opportunistic pathogens, as well as *Campylobacter spp, Klebsiella spp,Escherichia, and Salmonella* and its rise could be shown as a potential indicator of gut diseases. The *Firmicutes* abundance have been evidence to be positive relationship with energy and active transport, facilitated diffusion, endocytosis and passive diffusion, whereas improve in fecal *Proteobacteria and Bacteroidetes* is associated with inferior nutrient digestibility^2^. Therefore, higher abundances of the phylum *Firmicutes* along with lower abundances of the phylum *Proteobacteria* and *Bacteroidetes* might promote nutrient digestibility in weaning pigs. Eren et al.^53^ reported that the herbivores have an increase abundance of *Lachnospiraceae* than carnivores in animal. Vacca et al.^54^ reported that reduce abundance of *Lachnospiraceae,* while multiple sclerosis and ulcerative colitis patients. All participants of *Lachnospiraceae* are anaerobic, hemoorganotrophic and fermentative and could degrade non-starch polysaccharides and butyrate and acetic acid. Butyrate provide the major energy source for intestinal epithelial cell growth, increased intestinal protection mediated epithelial cells and favoring the suppression of inhibits inflammatory responses^55,56^. Stanley et al.^57^ showed that *Lachnospiraceae* is associated with improve growth performance in animals. The abundances of the genera *Lachnoclostridium*—butyric-acid-producing microbes that have been associated in the mitigation on intestinal inflammation- were better in the FSBM groups^10^. The *Lactobacillus* as a possibility probiotic, possess the opposition to pathogen, anti– inflammatory, antioxidant capacity, and capability to higher of fecal microbiota^58,59^. Zhu et al.^48^ reported an improved *Lactobacilli* and totality anaerobic bacteria counts in the gut microbiota weaning piglets due to dietary FSBM supplementation. In the current study, inclusion of FSBM treatments an increased in the abundances of the genera Lactobacillus were significantly increased RBC, WBC, Lymphocyte, glucose levels and decreased BUN. *Lactobacillus* is familiar to have a positive influence on the GIT, growth performance, and nutrient digestibility in pigs and regularly used as probiotics in livestock production^60,61^. Yan et al.^62^ reported that *Lactobacillus* could commonly increase growth performance and the GIT of animals by defense the intestine from pathogens and encourage efficient nutrient and energy extraction by the host. The genus *Prevotella* is saccharolytic and produce succinic and acetic acids as ending fermentation products^63^. *Prevotella* specialized in degrading fiber diet, which had also been correspondent with could improve intestinal immune and decrease diarrhea^64^. Wu et al.^65^ reported evidence indicated a close relationship among *Prevotella* and long period of time carbohydrates diets or carbohydrates from fiber-rich diets. It was also reported that a higher abundance of Prevotellaceae dominated in fecal microbiota of healthy piglets when compared to post-weaning diarrheic piglets 6^4^. Feng et al.^47^ and Forsyth et al.^66^ reported a higher *Prevotella* abundance, the higher the mucin composite content, but bacterial toxins would reduce gut penetrability and the sensitivity of the region-specific gut to systemic exposure. Accordingly, higher in the Prevotella abundances in FSBM it could be helpful in improve on growth performance in weaning piglets. The relative abundance of *Clostridium sensu stricto1* and *bacteoides* showed a positively correlated with the frequency of diarrhea. Clostridium is the main cause of diarrhoea in humans and is responsible for community-acquired out breaks^67^. Harlow et al.^68^ reported that *Clostridium perfringens, Clostridium difficile and Salmonella spp.* are the most common microbes associated with diarrhea. In our study, lower in the abundances of the genera *Clostridium sensu stricto1* in the FSBM group, and was positively correlated with decreased diarrhoea. Another result of FSBM groups were a decrease *Escherichia-Shigella*, and lower *Escherichia-Shigella* was main participant of the decreased in *Proteobacteria* abundance compared to the SBM treatments. The genera *Escherichia–Shigella* comprises an opportunistically pathogenic bacterium. Sousa et al.^69^ and Gong et al.^70^ reported the genera *Escherichia–Shigella* could destroy the gut structure and must be pro-inflammatory actions through multiple pathways and like the secretion of virulence factors, consequent in the improved danger of infection and diarrhea in the host. *Parabacteroides and Bacteroides,* which occur at the initial phase of the lifetime, have been informed to generate gamma amino butyric acid, closely related to growth^71,72^. A higher abundance of the genera *Bacteroides* is commonly used in the events of colorectal cancer, functional gastrointestinal disorders and ulcerative colitis^73–75^. Therefore, the biological results found that appropriate inclusion of FSBM in diets can inhibit the gut pathogens (such as *Escherichia–Shigella, Bacteroides and Parabacteroides*) and enhance beneficial bacteria (such as *Prevotella and Lactobacillus*) and further enhance the immunity and health status of weaning piglets.

## Conclusions

In conclusion, FSBM replacing SBM improved the growth performance, apparent total tract digestibility, blood profiles, possibly via altering gut microbiota profile in weaned piglets. The present study provides theoretical support for applying FSBM at 6 to 9% promote blood profiles and regulate intestinal health in weaning piglets.

## Data Availability

All data generated in this study are included in the published article. The datasets generated during the current study are available from the corresponding author on demand upon reasonable request.
